# Multimodal deep learning for predicting protein ubiquitination sites

**DOI:** 10.1093/bioadv/vbaf200

**Published:** 2025-08-20

**Authors:** Subash C Pakhrin, Moriah R Beck, Punjan Subedi, Rabina Lama, Simonsha Shrestha

**Affiliations:** School of Computing, Wichita State University, Wichita, KS 67260, United States; Department of Computer Science and Engineering Technology, University of Houston-Downtown, Houston, TX 77002, United States; Department of Chemistry and Biochemisty, Wichita State University, Wichita, KS 67260, United States; Department of Computer Science and Engineering Technology, University of Houston-Downtown, Houston, TX 77002, United States; Department of Computer Science and Engineering Technology, University of Houston-Downtown, Houston, TX 77002, United States; Department of Computer Science and Engineering Technology, University of Houston-Downtown, Houston, TX 77002, United States

## Abstract

**Motivation:**

Ubiquitination is a crucial post-translational modification that regulates various biological functions, including protein degradation, signal transduction, and cellular homeostasis. Accurate identification of ubiquitination sites is essential for understanding these mechanisms, yet existing prediction tools often lack generalizability across diverse datasets. To address this limitation, we developed Multimodal Ubiquitination Predictor, a deep learning-based approach capable of predicting ubiquitination sites across general, human-specific, and plant-specific datasets. By integrating diverse protein sequence representations—one-hot encoding, embeddings, and physicochemical properties—within a unified deep-learning framework, the proposed method significantly enhances prediction accuracy and robustness, offering a valuable resource for both research and applications in ubiquitination site discovery.

**Results:**

Multimodal Ubiquitination Predictor achieved superior performance across general, human-specific, and plant-specific datasets, with 77.25% accuracy, 74.98% sensitivity, 80.67% specificity, an MCC of 0.54, and an AUC of 0.87 on an independent human ubiquitination test dataset. It outperformed existing methods, demonstrating enhanced reliability for ubiquitination site prediction. This robust predictor and dataset serve as valuable resources for future research and discovery.

**Availability and implementation:**

The developed tool, programs, training, and test dataset are available at https://github.com/PakhrinLab/MMUbiPred.

## 1 Introduction

Ubiquitination is a reversible post-translational modification (PTM) where ubiquitin, a 76-amino acid protein discovered by Goldstein *et al.*, is attached to target proteins in eukaryotes (Goldstein *et al.* 1975, Wilkinson 2005). This process involves a complex interaction between the ubiquitin-activating enzyme (E1), ubiquitin-conjugating enzyme (E2), and ubiquitin ligases (E3) (Hershko and Ciechanover 1998). Ubiquitination plays a key role in protein degradation through the ubiquitin-proteasome pathway across all tissues (Lecker *et al.* 2006, Gu *et al.* 2023). It also regulates stem cell maintenance and differentiation by controlling pluripotency (Suresh *et al.* 2016). Additionally, ubiquitination is critical for various cellular functions, including proliferation, transcription regulation, deoxyribonucleic acid repair, replication, trafficking, virus budding, signal transduction, apoptosis, immune signaling, and autophagy (Karve and Cheema 2011, Alonso and Friedman 2013, Micel *et al.* 2013). Disruption of the ubiquitin pathway can lead to diseases like cancer, metabolic disorders, inflammatory conditions, t2 diabetes, and neurodegenerative diseases (Popovic *et al.* 2014, Foot *et al.* 2017). In the human proteome, ubiquitination is more prevalent in cytoskeletal, cell cycle, regulatory, and cancer-related proteins compared to other functional categories (Radivojac *et al.* 2010).

Mass spectrometry is commonly used to identify ubiquitination sites, these experimental methods are time-consuming and labor-intensive. As a result, computational tools utilizing machine learning (ML) and deep learning (DL) have become increasingly important for predicting ubiquitination sites. Several computational methods have been developed for this purpose. For instance, UbiPred uses a support vector machine (SVM) based on 31 physicochemical properties of amino acids (Tung and Ho 2008). UbPred, a random forest (RF)-based tool, predicts yeast ubiquitination sites using evolutionary data, amino acid composition, and physicochemical properties (Radivojac *et al.* 2010). UbiProber integrates key position and amino acid residue information using SVM based ML algorithm for general and species-specific ubiquitination site prediction (Chen *et al.* 2013). hCKSAAP_UbSite employs a stacking ensemble learning approach to predict human ubiquitination sites using multiple SVM classifiers trained with various feature types (Chen *et al.* 2013). iUbiq-Lys uses an SVM with evolutionary information to predict ubiquitination sites (Qiu *et al.* 2015). UbiSite combines multiple features such as amino acid composition (AAC), position-specific scoring matrices (PSSM), solvent-accessible surface area (SASA), and substrate motifs in a two-layer SVM model (Huang *et al.* 2016). ESA-UbiSite employs an evolutionary screening algorithm that identifies effective negative samples from non-validated sites to predict human ubiquitination sites (Wang *et al.* 2017). Collectively, these tools underscore the growing importance of feature engineering and model design in developing accurate computational predictors for ubiquitination sites.

Recently, several DL-based approaches have been proposed in the bioinformatics domain, with recent advancements focusing on predicting ubiquitination sites (Dai *et al.* 2018, 2024, Kong *et al.* 2020, Yang *et al.* 2020, 2022, Wang *et al.* 2021, Guo *et al.* 2023). DeepUbiquitylation employs a multimodal DL architecture, encoding ubiquitination and non-ubiquitination fragments using one-hot encoding, top 13 physicochemical properties, and evolutionary features, which are processed by three independent DL modules and fused at the decision level to predict general ubiquitination sites (He *et al.* 2018). DeepUbi uses sequential features with DL architecture for general ubiquitination site prediction (Fu *et al.* 2019). DeepTL-Ubi applies deep transfer learning methods to predict multispecies ubiquitination sites (Liu *et al.* 2021). Wang *et al.* developed a plant-specific predictor using word2vec and a 1D convolutional neural network (1D-CNN) (Wang *et al.* 2020). UbiComb utilizes a hybrid DL model combining convolutional neural networks and long short-term memory (LSTM) to predict plant ubiquitination sites (Siraj *et al.* 2021). HUbiPred predicts human ubiquitination PTMs by combining binary encoding and physicochemical properties of amino acids with two 1D-CNN and two LSTM models (Wang *et al.* 2021). Similarly, Caps-Ubi uses a hybrid encoding scheme with one-hot and amino acid continuous-type encodings in combination with 1D-CNN and capsule networks for multispecies ubiquitination site prediction (Luo *et al.* 2022). Mahdi *et al.* combined handcrafted features, such as position-specific scoring matrices, with raw protein sequences features and analysed them using LSTM networks (Pourmirzaei *et al.* 2023). This approach resulted in a modest enhancement of model performance. Shrestha *et al.* fine-tuned ProtGPT2 protein language model using a 21-window sequence of ubiquitinated and non-ubiquitinated lysine sites (where lysine is in the middle) along with their corresponding labels (Ferruz *et al.* 2022, Shrestha *et al.* 2024). While these methods have significantly improved ubiquitination site prediction, they often suffer from high false positive rates, especially in imbalanced datasets where negative samples far outnumber positive ones. Additionally, many DL approaches rely on predefined feature representations, which may limit generalizability across different species or biological contexts.

While there are existing predictors for general (non-organism-specific), human, and plant-specific ubiquitination sites, no generalized framework has been developed to predict ubiquitination sites across these three categories. Current predictors, though useful, are limited by small training datasets or the lack of availability of public datasets, shallow ML models, and varying performance. To overcome these challenges, a more advanced and generalized computational framework with a comprehensive ubiquitination dataset is required to predict ubiquitination sites across various species. Therefore, MMUbiPred (**M**ulti**m**odal **U**biquitination **P**redictor) was developed as a DL-based framework that incorporates multiple input modalities using three encoding schemes: embedding encoding, one-hot encoding, and physicochemical properties. The embedding encoding and one-hot encoding modules utilize 1D-CNNs to extract features, while the physicochemical properties encoding module uses LSTM to extract features (Hochreiter and Schmidhuber 1996, Kawashima *et al.* 2008, Mikolov *et al.* 2013, LeCun *et al.* 2015). The feature vectors produced from three sub-modules are concatenated and passed to a multi-layer perceptron (MLP) for deeper feature extraction and classification. Trained on general, plant, and human species datasets, and tested on independent test datasets from the same categories, MMUbiPred outperforms current state-of-the-art predictors in predicting ubiquitination sites.

## 2 Methods

### 2.1 Datasets

In this study, five ubiquitination datasets were used. The first dataset was sourced from the PLMD database (DeepUbi predictor used the same dataset), the largest online repository for protein lysine modifications in various eukaryotic and prokaryotic organisms (Xu *et al.* 2017). The original dataset contains 121 742 ubiquitination sites from 25 103 proteins. Initially, redundant protein sequences were removed using the psi-cd-hit software with a 30% sequence similarity cutoff, resulting in 12 038 unique and diverse proteins (Huang *et al.* 2010). From these 12 038 proteins, we identified 54 181 positive ubiquitination sites that were annotated in the PLMD database. A sequence window around the lysine (K) site of interest was created, including 24 residues upstream and downstream. For sites near the N-terminal or C-terminal, virtual amino acids (“-”) were concatenated to ensure the window size remained constant at 49. Lysine residues not annotated as ubiquitination sites in these proteins were considered as negative sites. The non-ubiquitination fragments were filtered to remove redundancy using a 30% sequence similarity cutoff, and random under-sampling was applied, yielding a final set of 50 170 negative samples. The dataset was then divided into training and independent test sets, with the independent test set containing no proteins or positive/negative ubiquitination sites that appear in the training set. Additionally, since the DeepUbi ubiquitination dataset is not publicly available, we undertook extensive efforts to reconstruct it by meticulously following the detailed methodology outlined in Fu *et al.*’s research (Fu *et al.* 2019). This reconstruction process ensured the dataset’s integrity and alignment with the DeepUbi study’s specifications. The protein and site counts are provided in Table 1.

**Table 1.** General ubiquitination sites from PLMD datasets (Xu *et al.* 2017).

Dataset	Proteins	Status	No. of sites	Total
Training	10 731	Positive	46 600	91 750
		Negative	45 150	
Independent test	1307	Positive	7581	12 601
		Negative	5020	

To further validate the findings from the PLMD database, we also examined the latest state-of-the-art CPLM 4.0 human ubiquitination PTM datasets (Xu *et al.* 2017, Zhang *et al.* 2022) Using the same approach as applied to the PLMD dataset, we extracted positive and negative training instances along with an independent test set. The detailed statistics of this dataset are present in Table 2.

**Table 2.** Human ubiquitination sites from CPLM 4.0 datasets (Zhang *et al.* 2022).

Dataset	Proteins	Status	No. of sites	Total
Training	7507	Positive	74 165	145 533
		Negative	71 368	
Independent test	835	Positive	8227	17 398
		Negative	9171	

To extend the validation of general ubiquitination findings (i.e. from the first dataset), we used the complementary general ubiquitination dataset from Shrestha *et al.* derived from dbPTM dataset (Li *et al.* 2022, Shrestha *et al.* 2024). The statistical details of the proteins, including the number of ubiquitination and non-ubiquitination sites in the training and testing sets, are provided in Table 3. Furthermore, to mitigate class imbalance in the training dataset (Shrestha *et al.* 2024), we employed a random under-sampling strategy. This technique was not applied to the independent test set in order to maintain its original distribution and integrity. The fourth dataset, derived from Chen *et al.*, includes ubiquitination sites from *Homo sapiens*, with the training and testing site counts shown in Table 4 (Chen *et al.* 2013). The fifth dataset, adapted from Siraj *et al.*, contains ubiquitination sites from various plant species, including *Arabidopsis thaliana*, *Oryza sativa Japonica*, and *Oryza sativa Indica* (Table 5) (Siraj *et al.* 2021). Additionally, sequence logos for (a) the general ubiquitination dataset (derived from PLMD dataset), (b) the plant ubiquitination dataset, and (c) the human ubiquitination datasets are provided in Supplementary Fig. S1, available as supplementary data at *Bioinformatics Advances* online (Crooks *et al.* 2004).

**Table 3.** General ubiquitination sites from Shrestha *et al.* (dbPTM) datasets (Shrestha *et al.* 2024).

Dataset	Proteins	Status	No. of sites	Total
Training	–	Positive	101 845	517 541
		Negative	415 696	
Independent test	2077	Positive	753	2077
		Negative	1324	

**Table 4.** Human ubiquitination sites obtained from the hCKSAAP_UbSite dataset (Chen *et al.* 2013).

Dataset	Proteins	Status	No. of sites	Total
Training	2500	Positive	6093	12 199
		Negative	6106	
Independent test	1352	Positive	3406	6809
		Negative	3403	

**Table 5.** Plant-specific ubiquitination sites extracted from the UbiComb dataset (Siraj *et al.* 2021).

Dataset	Proteins	Status	No. of sites	Total
Training	1739	Positive	2672	5410
		Negative	2738	
Independent test	412	Positive	743	1490
		Negative	747	

### 2.2 Feature encoding

MMUbiPred employs three types of feature encodings: one-hot encoding, supervised amino acid embeddings, and physicochemical properties. A concise overview of each encoding method is provided below:

One-hot encoding: The 49-sequence window surrounding the ubiquitination and non-ubiquitination lysine residues (lysine in the middle) was represented by one-hot encoding scheme. Each of the 21 distinct amino acids (20 canonical amino acids plus the pseudo-residue “-”) is encoded as a 21-dimensional feature vector. The amino acids are ordered as ARNDCQEGHILKMFPSTWYV-, where alanine (A) is encoded as (1,0,0,0,0,0,0,0,0,0,0,0,0,0,0,0,0,0,0,0,0) and cysteine (C) as (0,0,0,0,1,0,0,0,0,0,0,0,0,0,0,0,0,0,0,0,0), etc. Each peptide with a length of 49 is converted into a matrix with dimensions of 49 × 21.

Supervised amino acid embedding: In this embedding scheme, the model learns the representation of each amino acid from integer-encoded sequences through backpropagation (Pakhrin *et al.* 2023). To obtain the supervised amino acid embedding, the 20 canonical amino acids and one pseudo-residue (“-”) are first mapped to integers in the range from 0 to 20. These integers are then passed into the embedding layer, which initially contains random values. As training progresses through multiple epochs, the embedding layer refines the vector representations. Key parameters of the embedding layer include output_dim the size of the vector space and input_length the size of the input sequence. Consequently, the output from the embedding layer has dimensions of 49 × 21, where 49 represents the input peptide length and 21 is the output dimension. The one-hot and supervised amino acid embedding encodings effectively capture local sequence motifs, conserved patterns commonly recognized by E3 ligases during ubiquitination.

Encoding using physicochemical properties: In foundational work such as UbiPred, an Informative Physicochemical Property Mining Algorithm (IPMA) was applied to distill 31 key physicochemical properties from a pool of 531 (all drawn from the AAindex database, using main‐effect difference values to assess predictive importance) (Kawashima *et al.* 2008, Tung and Ho 2008). These 31 properties were then used to encode each amino acid into a 31-dimensional feature vector. We adopted the same encoding strategy for our 49-residue peptide window. Before encoding, each physicochemical attribute was min–max normalized to a [0, 1] range using a MinMaxScaler. As a result, each sequence fragment is represented as a 49 × 31 matrix, where 49 is the peptide length and 31 is the number of normalized features per amino acid.

Contextualized features from protein language models: Protein language models (PLMs) are utilized to capture the intricate biological patterns present in protein sequences (Littmann *et al.* 2021, Erckert *et al.* 2025). These models are constructed based on the transformer architecture, which integrates positional encoding and multi-head self-attention along with a parallelization scheme (Vaswani *et al.* 2017). Operating on a self-supervised learning framework, PLMs undergo training using extensive protein sequence corpora, enabling them to independently grasp the complex patterns and relationships within these sequences. Furthermore, the representations acquired by PLMs have wide-ranging applications in various downstream tasks, such as predicting contact maps, subcellular localization, long-range dependencies, protein structures, and deciphering protein function. Moreover, long-range dependency information is embedded in the PLM feature vectors, which are crucial for gaining insights into the broader context and functional implications of PTMs. These dependencies illuminate intricate connections between distant amino acids, thereby improving the accuracy of prediction algorithms. We utilized the feature vectors generated by three state-of-the-art protein language models: ProtT5-XL-UniRef50, which produces 1024-dimensional embeddings; ESM2-3B, which yields 2560-dimensional embeddings; and xTrimoPGLM-10B, which outputs 4352-dimensional embeddings (Elnaggar *et al.* 2022, Lin *et al.* 2023, Chen *et al.* 2025). These vectors, extracted for ubiquitinated and non-ubiquitinated sites from the full protein embedding files, were used to train basic artificial neural network models for ubiquitination site prediction.

**Figure 1.**
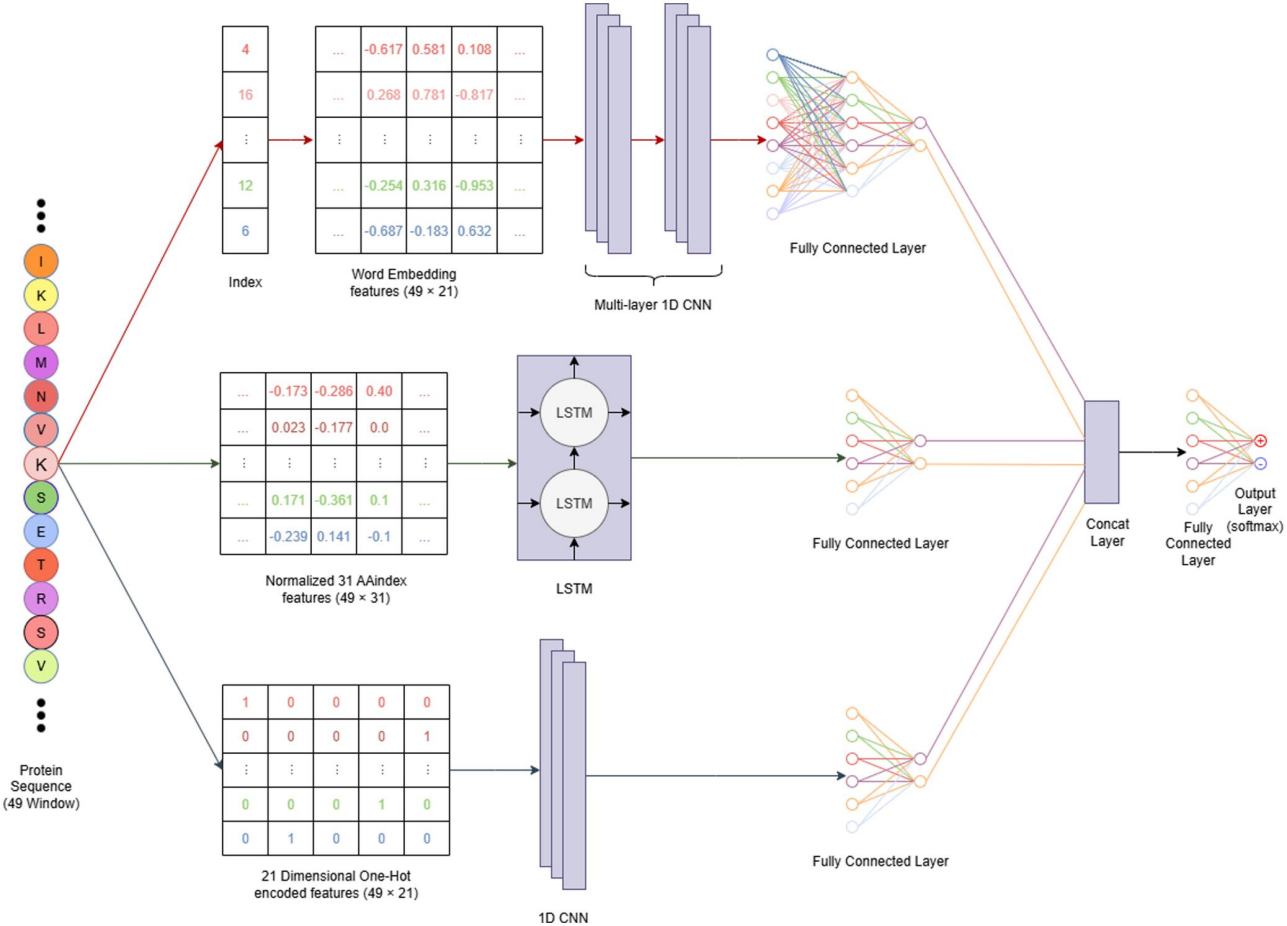
MMUbiPred processes protein sequences through three subnets that transform input features into a latent space, which are then concatenated and refined by a fully connected network to classify ubiquitination PTM sites.

Architecture of MMUbiPred: The MMUbiPred model is designed with three information processing subnets, each utilizing one of the three input feature encodings. These subnets transform protein sequences into a latent representative feature space through a series of nonlinear operations. Subsequently, a concatenation layer integrates the outputs from the three subnets, and the merged information is fed into a fully connected (FC) network. This network refines the combined latent feature vector and acts as the final classifier. The DL architecture of MMUbiPred is present in Fig. 1.

One-hot subnet: This subnet processes the one-hot encoded 49-residue ubiquitination sequence window using 1D-CNN layer. The 1D-CNN layer converts the sparse one-hot vector into a dense feature map that captures sequential patterns. The CNN layer uses a filter of size 3 and the ReLU activation function. The feature maps generated by the CNN layer are passed through a max pooling layer and then flattened into a one-dimensional (1D) feature vector. This vector is processed by a FC layer, and the output is further refined by an additional FC layer with two neurons to produce the final probability values.

Embedding subnet: The supervised amino acid embedding subnet takes an integer-encoded window sequence (size = 49) as input. This sequence is first processed through Keras’s embedding layer, followed by two 1D-CNN layers. The resulting feature maps from the 1D-CNN layers are flattened into a 1D vector, which is then passed to FC network with two hidden layers. The final FC layer outputs probability values. 1D-CNNs are essentially well-suited for detecting local features, as they learn motifs across sequences using spatially constrained filters.

Physicochemical subnet: The input to the physicochemical property subnet is a 49 × 31 matrix, where 49 represents the window size and 31 corresponds to the number of physicochemical properties. This matrix is normalized with min–max scaler before being fed into an LSTM layer composed of multiple LSTM units. Essentially, LSTMs were chosen to capture long-range dependencies in sequences, as physicochemical properties often exhibit position-dependent patterns that span beyond local receptive fields. These units are capable of learning long-term dependencies and addressing the vanishing gradient issues common in traditional recurrent neural networks. The output from the LSTM layer is flattened and passed through a FC layer to generate probability values.

Score-level fusion of the subnets: The probabilities generated by the three subnets are combined using a concatenation layer and subsequently passed through a FC layer with six neurons. This layer is followed by an output layer with two neurons and a SoftMax activation function. The architectures and associated hyperparameters for each subnet were determined through 10-fold cross-validation (CV) using a grid search strategy. Details of all hyperparameters used in MMUbiPred are provided in Supplementary Table S1, available as supplementary data at *Bioinformatics Advances* online. Additionally, a standard decision boundary of 0.5 was employed for model calibration.

Model evaluation and performance metrics: The model’s performance was evaluated using several metrics: Matthew’s correlation coefficient (MCC), sensitivity (SN)/recall (REC), specificity (SP), accuracy (ACC), precision (PRE), *F*1 score (F1), area under the precision–recall curve (PrAUC), and the area under the receiver operating characteristic curve (AUROC). A 10-fold CV was conducted on the benchmark training dataset, and the resulting model was further assessed using an independent test dataset.

ACC represents the proportion of correctly predicted residues out of the total residues (Eq. (1)). Sensitivity/recall quantifies the model’s ability to detect true positives among actual positives (Eq. (2)), while specificity (SP) measures its capacity to correctly classify negative residues (Eq. (3)). Precision (PRE) measures the proportion of correctly predicted positive observations to the total predicted positive observations (Eq. (4)). MCC provides a comprehensive evaluation of the model’s predictive performance for both positive and negative residues (Eq. (5)). The *F*1 score is the harmonic mean of precision and recall. It provides a balance between precision and recall, especially when there is an uneven class distribution (Eq. (6)). The ROC curve offers a visual depiction of the classifier’s diagnostic ability, and the area under the ROC curve (AUC) serves as a comparative measure across different models. A model with a higher AUC demonstrates superior classification performance compared to those with lower AUC values. The precision–recall curve plots precision against recall across varying thresholds. It highlights the tradeoff between precision and recall, offering insights into model performance, especially for imbalanced datasets where the positive class is of greater interest. The area under the precision–recall curve (AUPRC) summarizes this performance, with higher values indicating better ability to identify positives while minimizing false positives

(1) $$\mathrm{Accuracy}=\frac{\mathrm{TP}+\mathrm{TN}}{\mathrm{TP}+\mathrm{FN}+\mathrm{FP}+\mathrm{TN}}\times 100$$

 

(2) $$\mathrm{Sensitivity}/\mathrm{recall}=\frac{\mathrm{TP}}{\mathrm{TP}+\mathrm{FN}}\times 100$$

 

(3) $$\mathrm{Specificity}=\frac{\mathrm{TN}}{\mathrm{TN}+\mathrm{FP}}\times 100$$

 

(4) $$\mathrm{Precision}=\frac{\mathrm{TP}}{\mathrm{TP}+\mathrm{FP}}\times 100$$

 

(5) $$\mathrm{MCC}=\frac{\left(\mathrm{TP})(\mathrm{TN})-(\mathrm{FP})(\mathrm{FN}\right)}{\sqrt{\left(\mathrm{TP}+\mathrm{FP})(\mathrm{TP}+\mathrm{FN})(\mathrm{TN}+\mathrm{FP})(\mathrm{TN}+\mathrm{FN}\right)}}$$

 

(6) $$F1\;\mathrm{score}=2\times \frac{\mathrm{PRE}\times \mathrm{REC}}{\mathrm{PRE}+\mathrm{REC}}$$

## 3 Results

### 3.1 Optimal window size

**Figure 2.**
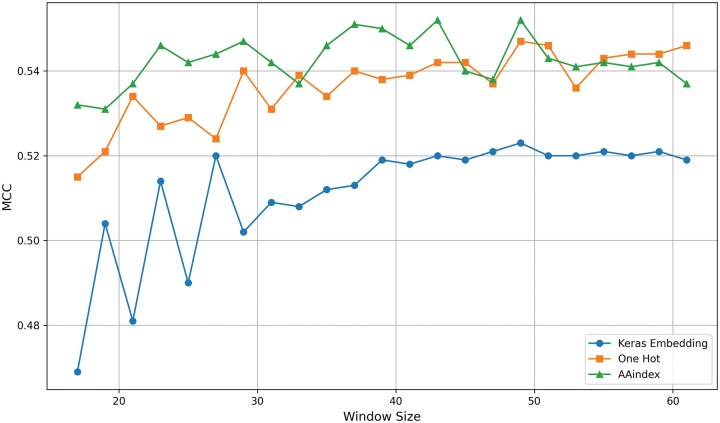
The highest mean MCC from 10-fold cross-validation on the ubiquitination training dataset was achieved with a window size of 49 and lysine (“K”) at the center for all encoding methods (one-hot, physicochemical, embeddings).

Ubiquitination status of an amino acid is influenced by its neighboring residues. Therefore, a window centered on the lysine residue, with an equal number of residues on both sides, is used as input for prediction tasks (Pakhrin *et al.* 2021, 2022, 2023). To identify the optimal window size, 10-fold CV was conducted using three encoding schemes—one-hot encoding, 31 informative physicochemical properties, and embedding encoding—on various window sizes ranging from 17 to 61 with an increment of two. This analysis utilized the general ubiquitination training dataset (Table 1) with the corresponding subnet architecture. The MCC for each window size and encoding is shown in Fig. 2, while additional performance metrics are provided in Supplementary Tables S2–S4, available as supplementary data at *Bioinformatics Advances* online.

From Fig. 2, it is evident that a window size of 49 yields the highest MCC for all three encodings: one-hot encoding, physicochemical properties, and embeddings. Consequently, a window size of 49 was selected as the optimal input for ubiquitination PTM prediction and used in subsequent analyses. Notably, the DeepUbiquitylation general ubiquitination prediction method also employed the same window size (He *et al.* 2018). Among the encodings, the physicochemical properties encoding achieved the highest MCC, followed by one-hot encoding and amino acid embedding.

### 3.2 10-fold CV on the general ubiquitination training dataset

To tune hyperparameters and evaluate the performance of different model architectures, 10-fold CV was conducted on the general ubiquitination training dataset. Table 6 summarizes the predictive performance of various architectures utilizing three encoding schemes on the 10-fold CV. The results indicate that the architecture employing multimodal score-level fusion, which aggregates latent representations from the three encoding schemes, achieved the highest MCC of 0.5568 during stratified 10-fold CV on the general ubiquitination training dataset. The multimodal DL architecture achieved an MCC of 0.5568 ± 0.0086, an ACC of 77.65 ± 0.0060, a SN of 73.72 ± 0.0361, and a SP of 81.71 ± 0.0277. Performance metrics for the individual subnets, their combinations and the multimodal architecture are also present in Table 6. The results indicate that all the models with different encoding schemes exhibit a low standard deviation, suggesting a good fit and stability. Notably, the multimodal score-level fusion architecture, MMUbiPred, outperformed the three individual subnet architectures and their combinations in terms of MCC and ACC (provides modest results for SN and SP) for ubiquitination PTM prediction. Consequently, this multimodal DL architecture, comprising three subnets followed by FC layers, was selected to predict ubiquitination sites on an independent test dataset.

**Table 6.** Results of the 10-fold cross-validation on the general ubiquitination training dataset.

Encoding	MCC	SN	SP	ACC
PSSM	0.277 ± 0.003	65.8 ± 0.043	61.7 ± 0.043	63.8 ± 0.00
ESM-2 (3B)	0.436 ± 0.021	73.5 ± 0.013	70.0 ± 0.025	71.8±0.011
ProtT5	0.440 ± 0.041	71.7 ± 0.031	72.3 ± 0.020	72.0 ± 0.02
xTrimoPGLM-10B	0.453 ± 0.022	72.7 ± 0.028	72.5 ± 0.040	72.6 ± 0.011
One hot (OH)	0.547 ± 0.006	68.2 ± 0.055	85.3 ± 0.051	76.8 ± 0.003
Embedding (Em)	0.523 ± 0.010	**75.3 ± 0.018**	76.9 ± 0.018	76.1 ± 0.005
AAindex (AA)	0.552 ± 0.011	67.9 ± 0.031	86.2 ± 0.029	77.1 ± 0.005
AA + OH	0.551 ± 0.008	66.9 ± 0.005	**87.0 ± 0.040**	76.7 ± 0.081
AA + Em	0.552 ± 0.022	71.3 ± 0.059	83.1 ± 0.071	77.1 ± 0.010
OH + Em	0.538 ± 0.025	71.9 ± 0.075	81.0 ± 0.085	76.4 ± 0.011
MMUbiPred	**0.556 ± 0.008**	73.7 ± 0.036	81.7 ± 0.027	**77.6 ± 0.006**

The highest value in each column is emphasized in bold.

To leave no stone unturned as well as to test the utility of PLM’s contextualized embeddings of ubiquitinated and non-ubiquitinated sites, we further experimented with site-specific contextualized embeddings of ubiquitinated, and non-ubiquitinated lysine residues generated by ProtT5-XL-UniRef50, ESM2-3B, xTrimoPGLM-10B PLM (Elnaggar *et al.* 2022, Lin *et al.* 2023, Pakhrin *et al.* 2023, 2024, Palacios *et al.* 2024, Chen *et al.* 2025). Additionally, we extracted the 49-window PSSM features produced through PSI-BLAST (Altschul *et al.* 1997). Each amino acid was represented by 20 features, resulting in the extraction of a 49 × 20 matrix using the PSSM encoding scheme. In a PSSM, positive values indicate an increased likelihood of an amino acid occurring at a given position, reflecting evolutionary conservation, while zero values suggest neutrality. Negative values indicate that the amino acid is less likely to appear at that position, often due to unfavorable physicochemical properties or evolutionary constraints. However, the 10-fold CV results using the contextualized embeddings from the ProtT5, ESM2-3B, xTrimoPGLM-10B PLMs, and the PSSM features were subpar, leading to their exclusion from our experiments (Table 6). Furthermore, the features produced by the one-hot, embedding, and physicochemical subnet models from intermediate layers were concatenated and fed into a newly constructed MLP. This trained MLP was then evaluated using features extracted from an independent test dataset (see Table 6). Although the performance was promising, it was slightly lower than that of the MMUbiPred method.

### 3.3 Independent test performance for general ubiquitination

The MMUbiPred model was trained on the complete general ubiquitination training dataset (90% training and 10% validation) using hyperparameters obtained through a 10-fold CV grid search. The trained model was evaluated with PLMD general ubiquitination-independent test dataset (see Table 1) (Xu *et al.* 2017). MMUbiPred achieved an MCC of 0.5458, SN of 74.98%, SP of 80.67%, and ACC of 77.25%. The model classified 4050 samples as true negatives, 5682 as true positives, 970 as false positives, and 1896 as false negatives.

**Figure 3.**
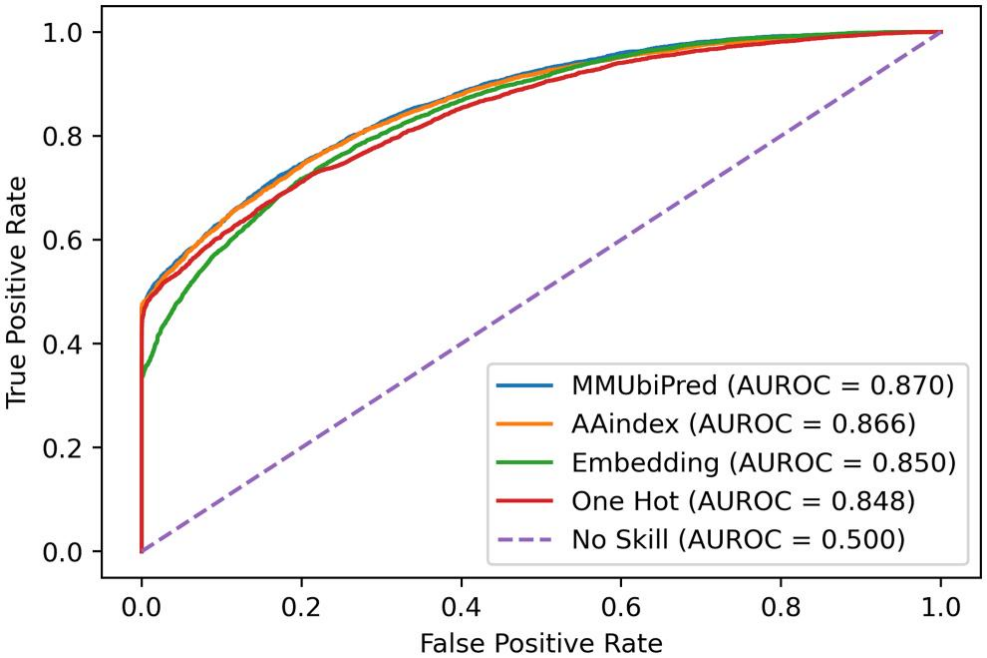
The ROC curve for the general PLMD ubiquitination independent test dataset shows MMUbiPred outperforming one-hot encoding, AAindex, and embedding-based DL with a higher AUROC, indicating better differentiation between ubiquitinated classes.

**Figure 4.**
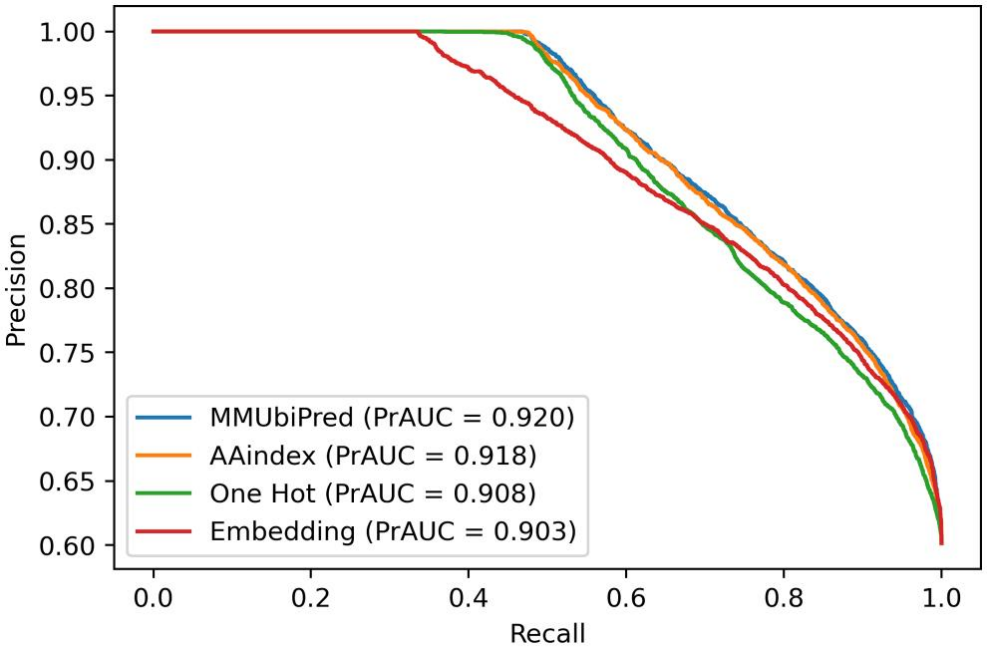
The precision–recall curve for the general PLMD ubiquitination-independent test dataset shows MMUbiPred outperforming one-hot, physicochemical, and embedding encoding schemes with a higher AUPRC, demonstrating better detection of true positives and fewer false positives in an imbalanced dataset.

Performance metrics for the individual subnets corresponding to the three encoding schemes and their combinations are detailed in Table 7, which highlights that MMUbiPred achieved the highest MCC. Additionally, the model attained the highest area under the curve (AUC) of 0.870, as depicted in Fig. 3, and the highest precision–recall area under the curve (AUPRC) of 0.920, as shown in Fig. 4. It should be noted that a higher AUC reflects the model’s strong ability to distinguish between positive and negative classes across various thresholds, while a higher AUPRC is especially beneficial for imbalanced datasets, as it ensures better precision–recall tradeoffs by accurately identifying true positives while minimizing false positives. Since DeepUbi utilized the same dataset, we compared the precision–recall area under the curve (PrAUC) of MMUbiPred and DeepUbi. MMUbiPred achieved a superior PrAUC of 0.919, surpassing DeepUbi’s 0.894. This indicates that our developed model consistently attains higher precision (fewer false positives) and higher recall (fewer false negatives). The AUPRC and AUROC curves comparing MMUbiPred with DeepUbi are present in Supplementary Fig. S2, available as supplementary data at *Bioinformatics Advances* online.

**Table 7.** Results of the general ubiquitination independent test dataset.

Encoding	MCC	SN	SP	ACC
One hot (OH)	0.517	68.4	84.3	74.7
Embedding (Em)	0.510	77.4	74.1	76.1
Concatenation	0.517	83.5	67.2	76.0
AAindex (AA)	0.530	72.7	81.3	76.1
AA + OH	0.539	73.6	81.4	76.7
AA + Em	0.541	74.2	80.9	76.9
OH + Em	0.539	67.3	**87.4**	75.3
MMUbiPred	**0.545**	**74.9**	80.6	**77.2**

The highest value in each column is highlighted in bold.

### 3.4 Independent test performance on CPLM 4.0 human ubiquitination

The MMUbiPred model was subjected to training on the entire human ubiquitination dataset from CPLM 4.0 (Zhang *et al.* 2022), post processed with a 30% PSI-CD-HIT filtering, employing a 90% training and 10% validation split. Optimal hyperparameters were meticulously derived from a comprehensive 10-fold CV grid search. The model’s capabilities were subsequently evaluated on a CPLM 4.0 independent human ubiquitination test dataset (see Table 2). MMUbiPred produced a strong MCC of 0.6232, SN of 75.68%, SP of 86.13%, and an ACC of 81.19%. It decisively classified 7897 instances as true negatives, 6226 as true positives, 1271 as false positives, and 2000 as false negatives. Supplementary Table S5, available as supplementary data at *Bioinformatics Advances* online illustrates the relative contributions of the three feature types—one-hot encoding, embeddings, and physicochemical properties—and their combinations to predictive performance on the human ubiquitination independent test dataset sourced from CPLM 4.0.

### 3.5 Comparisons with existing models

We conducted comprehensive comparisons with existing methods in four different benchmark datasets (Tables 8 and 9, Supplementary Tables S6 and S7, available as supplementary data at *Bioinformatics Advances* online). For each dataset, the MMUbiPred architecture was trained on the corresponding training set. Initially, the performance of MMUbiPred was compared to the DeepUbi method using the independent test set from the benchmark dataset, as detailed in Section 2 (Table 1). DeepUbi is a DL framework built on the inception model (Szegedy *et al.* 2015, Fu *et al.* 2019). While its source code is available, the dataset it uses is not. However, we have made our best effort to reconstruct the dataset as outlined in their research work. The general ubiquitination training dataset derived from DeepUbi research work was used to train the MMUbiPred model, and its performance was evaluated on the general ubiquitination independent test dataset. DeepUbi achieved an MCC of 0.4611 on the general ubiquitination independent test dataset, while MMUbiPred achieved an MCC of 0.5458 (Table 8).

**Table 8.** Predictive performance of MMUbiPred compared to DeepUbi model.

Predictors	MCC	SN	SP	ACC
MMUbiPred	**0.5458**	**74.98**	**80.67**	**77.25**
DeepUbi	0.4611	72.70	74.24	73.31

The highest value in each column is emphasized in bold.

**Table 9.** Predictive performance of MMUbiPred compared to Ubiq-PTMGPT2 model and various encoding schemes.

Predictors	MCC	F1	PRE	REC
MMUbiPred	**0.4415**	**0.668**	54.70	**85.90**
Embedding (Em)	0.4031	0.649	53.29	82.85
One hot (OH)	0.4114	0.649	56.39	76.33
AAindex (AA)	0.4125	0.647	57.11	74.73
AA + Em	0.4133	0.648	57.01	75.13
AA + OH	0.4139	0.654	53.31	84.71
OH + Em	0.3927	0.636	56.17	73.27
Ubiq-PTMGPT2	0.3125	0.357	**80.46**	22.97
Musite-Web	0.2624	0.276	75.67	16.93
DL-Ubiq	0.1091	0.333	36.67	23.76

The highest value in each column is emphasized in bold.

To validate our findings from the general ubiquitination dataset derived from DeepUbi research work, we conducted further experiments using the latest dbPTM ubiquitination dataset, as utilized by Shrestha *et al.* in their study (Li *et al.* 2022, Shrestha *et al.* 2024). For consistency, we employed the exact same training and independent test datasets used by Shrestha *et al.* for ubiquitination prediction tasks. MMUbiPred demonstrated significantly improved performance, achieving an MCC of 0.4134, precision of 0.5527, recall of 0.7952, and an *F*1 score of 0.6521 (see Table 9). These results are better than combination of various encoding schemes and Shrestha *et al.* research work, who fine-tuned ProtGPT2 using a 21-window sequence of ubiquitinated and non-modified sites along with their corresponding labels (Ferruz *et al.* 2022). This improvement aligns with the “no free lunch theorem”, which posits that no single model or algorithm performs best across all tasks or datasets (Wolpert and Macready 1997). While Shrestha *et al.* fine-tuned ProtGPT2, MMUbiPred’s specialized multimodal methodology better leveraged the specific patterns in the dbPTM ubiquitination dataset, resulting in superior performance (Li *et al.* 2022).

These findings highlight that ubiquitination is a complex and diverse PTM with unique characteristics that are not fully captured by the generalized contextualized embeddings of PLMs. While PLMs, trained on large-scale protein datasets, excel at learning broad contextual features applicable to various biological tasks, they often overlook the specific physicochemical properties crucial for ubiquitination. In other words, contextualized embeddings from foundation models like ProtGPT2, ESM2-3B, xTrimoPGLM-10B, and ProtT5 are proficient at capturing the long-term context of many biological mechanisms but lack the detailed understanding of the intricate nature of ubiquitination PTMs (Elnaggar *et al.* 2022, Ferruz *et al.* 2022, Lin *et al.* 2023, Chen *et al.* 2025). These results suggest that sequential and physicochemical features are the key determinants for accurate ubiquitination PTM prediction, outperforming the contextualized embeddings generated by PLMs.

Next, the performance of MMUbiPred in predicting plant-specific ubiquitination sites was compared with established approaches. The plant-specific ubiquitination dataset was sourced from Siraj *et al.* MMUbiPred was trained on the plant ubiquitination training dataset and evaluated using the plant ubiquitination independent test dataset (Siraj *et al.* 2021). The benchmark dataset for plant-specific ubiquitination is illustrated in the methods section (Table 5). The MMUbiPred architecture achieved an ACC of 84.56%, SP of 83.40%, SN of 85.73%, and an MCC of 0.6914. The results for UbPred, iUbiq-Lys, Ubisite, Deep Ubiquitylation, DeepUbi, and Wang *et al.* were adapted from UbiComb. Moreover, we utilized the exact same dataset as employed by UbiComb. Supplementary Table S6, available as supplementary data at *Bioinformatics Advances* online demonstrates that MMUbiPred outperforms all other prominent predictors on the plant-specific independent test dataset. MMUbiPred clearly surpasses UbiComb by leveraging a substantially richer physicochemical feature set and broader sequence context. Specifically, MMUbiPred uses 31 informative physicochemical properties, in contrast to UbiComb’s five-feature set (hydrophobicity, size, number of degenerate triplet codons, preference for β-strand formation, and frequency of occurrence in β-strands). Additionally, MMUbiPred applies these physicochemical features via a 49-residue window alongside one-hot encoding and embeddings, whereas UbiComb is confined to a 31-residue window, using only embeddings and the limited physicochemical feature set. Together, the richer feature representation and extended context allow MMUbiPred to deliver superior ubiquitination predictive performance.

Lastly, the human ubiquitination dataset was obtained from Chen *et al.* (2013). The MMUbiPred architecture was trained on the human ubiquitination training dataset, and the trained model was evaluated using the human ubiquitination independent test dataset. It achieved an MCC of 0.52, ACC of 75.90%, SN of 80.40%, and SP of 71.39% on independent human dataset respectively. A brief explanation of human-specific ubiquitination dataset is provided in Section 2 (Table 5). The hCKSAAP_UbSite model uses only the AUC to assess performance. MMUbiPred model achieves an AUC of 0.8248 on the independent test dataset, while the hCKSAAP_UbSite model has an AUC of 0.757 (see Supplementary Table S7, available as supplementary data at *Bioinformatics Advances* online). These findings suggest that the proposed MMUbiPred model provides more reliable and accurate predictions compared to existing methods for ubiquitination site prediction across different benchmark datasets.

### 3.6 t-Distributed stochastic neighbor embedding plot

**Figure 5.**
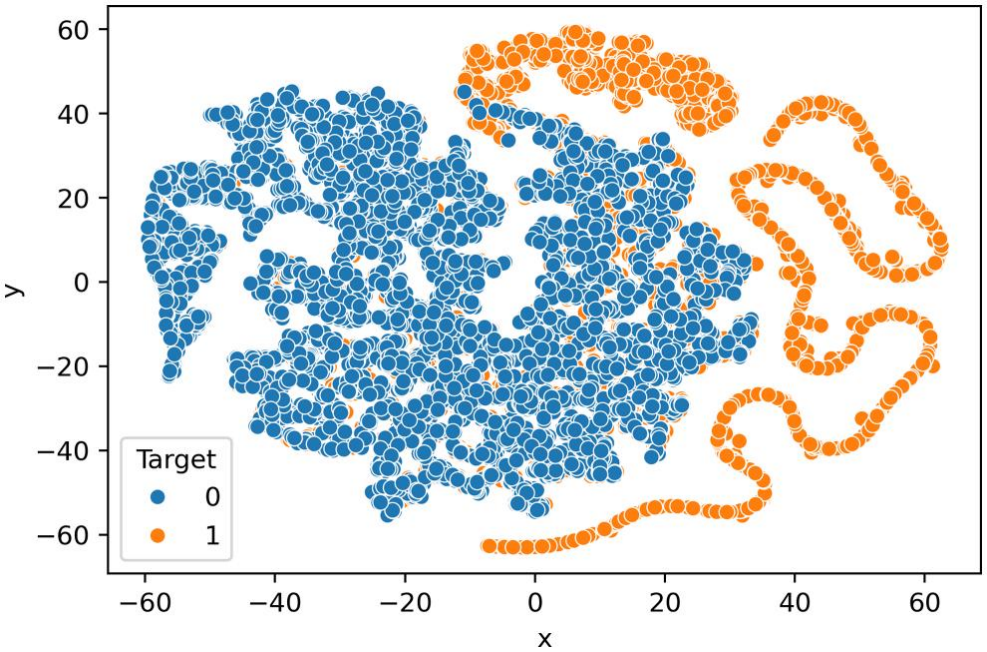
The t-SNE plot of the MMUbiPred trained model shows clear clustering of positive and negative ubiquitinated data, with samples on the left representing negative sites and those on the right representing positive ones.

The t-SNE technique was employed to visualize the classification efficacy of embeddings extracted from the penultimate dense layer of the MMUbiPred model (Maaten and Hinton 2008). This approach projects these features into a 2D space to visualize class boundaries. A learning rate of 50 was used for t-SNE to generate scatter plots for feature vectors produced from the penultimate FC layer of the trained MMUbiPred framework. The plots were created using the general ubiquitination-independent test dataset (Table 1), consisting of 5020 negative and 7581 positive samples. Ubiquitination PTM sites are often embedded within specific sequence motifs that are recognized by E3 ligases. These motifs include conserved amino acids and distinct physicochemical properties, such as charge, hydrophobicity, or secondary structure propensity (Radivojac *et al.* 2010, Qiu *et al.* 2015, Huang *et al.* 2016). These influence lysine accessibility and the probability of ubiquitination. t-SNE visualizations show clear clustering of positive (ubiquitinated) versus negative (non-ubiquitinated) samples in the 2D embedding space. This indicates that embeddings derived from the 49-residue window around target lysines encode biologically meaningful patterns reflective of known ubiquitination determinants. The pronounced separation seen in the plots underscores the model’s ability to learn complex sequence-based and physicochemical signatures, and accurately classify ubiquitination sites in reduced dimensionality (see Fig. 5).

## 4 Discussion

Three types of ubiquitination datasets were utilized to train the MMUbiPred: general ubiquitination, plant-specific ubiquitination, and human-specific ubiquitination datasets. MMUbiPred is a multimodal, score-fusion-based framework comprising three subnets, each representing distinct encoding schemes: one-hot encoding, physicochemical properties, and supervised embedding. The one-hot encoding subnet utilizes a 1D-CNN followed by a FC layer. Similarly, the supervised embedding subnet integrates a 1D-CNN followed by a FC layer. The physicochemical encoding subnet, however, employs an LSTM followed by a FC layer. The probability scores from these three subnets are fused and passed through a final FC layer to generate predictions.

The species-specific prediction approach used in MMUbiPred is crucial because ubiquitination systems differ significantly across species, primarily due to variations in ubiquitination-related enzymes such as E1 activating enzymes, E2 conjugating enzymes, and E3 ligases. These enzymes are vital for substrate recognition and modification, leading to species-specific ubiquitination patterns. By developing MMUbiPred models that are tailored to individual species, the accuracy of identifying ubiquitination sites is greatly enhanced. The Ubiquitin and Ubiquitin-like Conjugation Database serves as a valuable resource, providing detailed information on ubiquitination-related enzymes across diverse species (Gao *et al.* 2013). This highlights the inherent differences in ubiquitination mechanisms and underscores the importance of employing species-specific prediction tools.

In conclusion, MMUbiPred is a robust and versatile ubiquitination site prediction framework that outperforms existing methods across three different dataset types: general, plant-specific, and human-specific ubiquitination. MMUbiPred yields accurate and reliable predictions. MMUbiPred serves as a powerful tool for researchers to effectively identify ubiquitination sites in protein sequences, contributing to a deeper understanding of ubiquitination mechanisms.

## Supplementary Material

vbaf200_Supplementary_Data
