# Supplementary material for: Multimodal deep learning for predicting protein ubiquitination sites
Source: Bioinform Adv. 2025 Aug 20;5(1):vbaf200. doi: 10.1093/bioadv/vbaf200 (PMC12408473; doi:10.1093/bioadv/vbaf200)
Supplement: vbaf200_Supplementary_Data [file vbaf200_supplementary_data.docx]

**Multimodal deep learning for predicting protein ubiquitination sites**

Subash C. Pakhrin ^1, 2 *^, Moriah R. Beck ^3^, Punjan Subedi ^2^, Rabina Lama ^2^, Simonsha Shrestha ^2^

^1^School of Computing, Wichita State University, 1845 Fairmount St., Wichita, KS 67260, USA

^2^Department of Computer Science and Engineering Technology, University of Houston-Downtown, 1 Main St., Houston, TX 77002, USA

^3^Department of Chemistry and Biochemistry, Wichita State University, 1845 Fairmount St., Wichita, KS 67260, USA

Supplementary Table S1. The hyper-parameters of MMUbiPred model.

| Subnet | Layer | Hyper-parameters | | | | |
| --- | --- | --- | --- | --- | --- | --- |
|  |  | Activation Function | Size | Filters | Dropout | MaxPool 1D |
| Embedding  Encoding | 1D CNN | ReLU | 3 | 256 | 0.4 | Size (2) |
|  |  | ReLU | 7 | 256 | 0.4 | Size (2) |
|  | Flatten | - | - | - | - | - |
|  | Dense | ReLU | 768 | - | 0.5 | - |
|  |  | ReLU | 256 | - | 0.5 | - |
|  |  | SoftMax | 2 | - | - | - |
| One hot encoding | 1D CNN | ReLU | 3 | 16 | - | Size (2) |
|  | Flatten | - | - | - | - | - |
|  | Dense | ReLU | 512 | - | 0.5 | - |
|  |  | SoftMax | 2 | - | - | - |
| AAindex encoding | LSTM | ReLU | 16 | - | - | - |
|  | Flatten | - | - | - | - | - |
|  | Dense | ReLU | 512 | - | 0.5 | - |
|  |  | SoftMax | 2 | - | - | - |
| Multi-Layer  Perceptron | Dense | ReLU | 6 | - | - | - |
|  |  | SoftMax | 2 | - | - | - |

Supplementary Table S2. 10-fold cross-validation results for various window sizes in general ubiquitination dataset using the physicochemical modality

| **Window Size** | **MCC ± S.D.** | **SN ± S.D.** | **SP ± S.D.** | **ACC ± S.D.** |
| --- | --- | --- | --- | --- |
| 61 | 0.537 ± 0.015 | 0.711 ± 0.071 | 0.816 ± 0.078 | 0.764 ± 0.007 |
| 59 | 0.542 ± 0.011 | 0.693 ± 0.061 | 0.838 ± 0.061 | 0.766 ± 0.004 |
| 57 | 0.541 ± 0.006 | 0.708 ± 0.051 | 0.826 ± 0.049 | 0.767 ± 0.003 |
| 55 | 0.542 ± 0.011 | 0.693 ± 0.061 | 0.838 ± 0.061 | 0.766 ± 0.004 |
| 53 | 0.541 ± 0.006 | 0.708 ± 0.051 | 0.826 ± 0.049 | 0.767 ± 0.003 |
| 51 | 0.543 ± 0.012 | 0.706 ± 0.024 | 0.831 ± 0.029 | 0.769 ± 0.005 |
| 49 | 0.552 ± 0.011 | 0.679 ± 0.031 | 0.862 ± 0.029 | 0.771 ± 0.005 |
| 47 | 0.538 ± 0.020 | 0.696 ± 0.063 | 0.832 ± 0.069 | 0.764 ± 0.009 |
| 45 | 0.540 ± 0.014 | 0.694 ± 0.062 | 0.835 ± 0.062 | 0.765 ± 0.006 |
| 43 | 0.552 ± 0.009 | 0.737 ± 0.018 | 0.813 ± 0.020 | 0.775 ± 0.004 |
| 41 | 0.546 ± 0.010 | 0.636 ± 0.035 | 0.890 ± 0.033 | 0.763 ± 0.003 |
| 39 | 0.550 ± 0.009 | 0.702 ± 0.029 | 0.840 ± 0.031 | 0.771 ± 0.003 |
| 37 | 0.551 ± 0.005 | 0.716 ± 0.035 | 0.829 ± 0.030 | 0.773 ± 0.004 |
| 35 | 0.546 ± 0.010 | 0.625 ± 0.030 | 0.899 ± 0.027 | 0.762 ± 0.004 |
| 33 | 0.537 ± 0.012 | 0.642 ± 0.029 | 0.879 ± 0.029 | 0.760 ± 0.005 |
| 31 | 0.542 ± 0.009 | 0.704 ± 0.031 | 0.832 ± 0.033 | 0.768 ± 0.003 |
| 29 | 0.547 ± 0.012 | 0.682 ± 0.025 | 0.856 ± 0.029 | 0.769 ± 0.005 |
| 27 | 0.544 ± 0.009 | 0.683 ± 0.036 | 0.851 ± 0.037 | 0.767 ± 0.003 |
| 25 | 0.542 ± 0.009 | 0.670 ± 0.036 | 0.860 ± 0.031 | 0.765 ± 0.004 |
| 23 | 0.546 ± 0.013 | 0.631 ± 0.016 | 0.895 ± 0.018 | 0.763 ± 0.005 |
| 21 | 0.537 ± 0.012 | 0.642 ± 0.029 | 0.879 ± 0.029 | 0.760 ± 0.005 |
| 19 | 0.531 ± 0.012 | 0.663 ± 0.055 | 0.854 ± 0.053 | 0.759 ± 0.004 |
| 17 | 0.532 ± 0.012 | 0.644 ± 0.046 | 0.871 ± 0.043 | 0.758 ± 0.005 |

Supplementary Table S3. 10-fold cross-validation results for various window sizes in general ubiquitination dataset using one hot modality.

| **Window Size** | **MCC ± S.D.** | **SN ± S.D.** | **SP ± S.D.** | **ACC ± S.D.** |
| --- | --- | --- | --- | --- |
| 61 | 0.546 ± 0.010 | 0.660 ± 0.067 | 0.868 ± 0.060 | 0.764 ± 0.006 |
| 59 | 0.544 ± 0.011 | 0.706 ± 0.068 | 0.828 ± 0.066 | 0.767 ± 0.003 |
| 57 | 0.544 ± 0.010 | 0.673 ± 0.076 | 0.856 ± 0.064 | 0.764 ± 0.008 |
| 55 | 0.543 ± 0.013 | 0.684 ± 0.061 | 0.846 ± 0.059 | 0.765 ± 0.006 |
| 53 | 0.536 ± 0.013 | 0.728 ± 0.043 | 0.804 ± 0.045 | 0.766 ± 0.006 |
| 51 | 0.546 ± 0.013 | 0.678 ± 0.061 | 0.855 ± 0.059 | 0.766 ± 0.005 |
| 49 | 0.547 ± 0.006 | 0.682 ± 0.055 | 0.853 ± 0.051 | 0.768 ± 0.003 |
| 47 | 0.537 ± 0.018 | 0.684 ± 0.084 | 0.838 ± 0.085 | 0.761 ± 0.006 |
| 45 | 0.542 ± 0.009 | 0.691 ± 0.058 | 0.841 ± 0.051 | 0.766 ± 0.005 |
| 43 | 0.542 ± 0.015 | 0.668 ± 0.060 | 0.860 ± 0.060 | 0.764 ± 0.005 |
| 41 | 0.539 ± 0.010 | 0.701 ± 0.045 | 0.832 ± 0.041 | 0.766 ± 0.005 |
| 39 | 0.538 ± 0.012 | 0.662 ± 0.079 | 0.858 ± 0.075 | 0.760 ± 0.006 |
| 37 | 0.540 ± 0.008 | 0.658 ± 0.060 | 0.865 ± 0.057 | 0.762 ± 0.004 |
| 35 | 0.534 ± 0.013 | 0.683 ± 0.058 | 0.840 ± 0.061 | 0.762 ± 0.006 |
| 33 | 0.539 ± 0.011 | 0.660 ± 0.071 | 0.862 ± 0.064 | 0.761 ± 0.005 |
| 31 | 0.531 ± 0.023 | 0.662 ± 0.077 | 0.851 ± 0.083 | 0.757 ± 0.007 |
| 29 | 0.540 ± 0.015 | 0.607 ± 0.037 | 0.906 ± 0.034 | 0.757 ± 0.005 |
| 27 | 0.524 ± 0.017 | 0.653 ± 0.055 | 0.856 ± 0.058 | 0.755 ± 0.005 |
| 25 | 0.529 ± 0.018 | 0.636 ± 0.063 | 0.872 ± 0.063 | 0.754 ± 0.003 |
| 23 | 0.527 ± 0.022 | 0.626 ± 0.068 | 0.878 ± 0.067 | 0.752 ± 0.004 |
| 21 | 0.534 ± 0.019 | 0.586 ± 0.064 | 0.913 ± 0.060 | 0.749 ± 0.003 |
| 19 | 0.521 ± 0.025 | 0.606 ± 0.061 | 0.888 ± 0.063 | 0.747 ± 0.003 |
| 17 | 0.515 ± 0.030 | 0.609 ± 0.066 | 0.879 ± 0.072 | 0.744 ± 0.005 |

Supplementary Table S4. 10-fold cross-validation results for various window sizes in general ubiquitination dataset using the embedding modality.

| **Window Size** | **MCC ± S.D.** | **SN ± S.D.** | **SP ± S.D.** | **ACC ± S.D.** |
| --- | --- | --- | --- | --- |
| 61 | 0.519 ± 0.006 | 0.732 ± 0.021 | 0.786 ± 0.021 | 0.759 ± 0.003 |
| 59 | 0.521 ± 0.005 | 0.744 ± 0.028 | 0.775 ± 0.028 | 0.760 ± 0.002 |
| 57 | 0.520 ± 0.010 | 0.738 ± 0.023 | 0.780 ± 0.025 | 0.759 ± 0.004 |
| 55 | 0.521 ± 0.008 | 0.732 ± 0.028 | 0.787 ± 0.025 | 0.759 ± 0.004 |
| 53 | 0.520 ± 0.004 | 0.757 ± 0.013 | 0.762 ± 0.016 | 0.760 ± 0.002 |
| 51 | 0.520 ± 0.005 | 0.755 ± 0.023 | 0.764 ± 0.022 | 0.759 ± 0.002 |
| 49 | 0.523 ± 0.010 | 0.753 ± 0.018 | 0.769 ± 0.018 | 0.761 ± 0.005 |
| 47 | 0.521 ± 0.011 | 0.737 ± 0.027 | 0.782 ± 0.029 | 0.760 ± 0.005 |
| 45 | 0.519 ± 0.008 | 0.741 ± 0.026 | 0.776 ± 0.026 | 0.759 ± 0.004 |
| 43 | 0.520 ± 0.010 | 0.748 ± 0.018 | 0.771 ± 0.022 | 0.760 ± 0.005 |
| 41 | 0.518 ± 0.005 | 0.743 ± 0.019 | 0.774 ± 0.017 | 0.758 ± 0.002 |
| 39 | 0.519 ± 0.008 | 0.739 ± 0.027 | 0.778 ± 0.029 | 0.759 ± 0.003 |
| 37 | 0.513 ± 0.009 | 0.741 ± 0.027 | 0.770 ± 0.025 | 0.756 ± 0.004 |
| 35 | 0.512 ± 0.010 | 0.753 ± 0.038 | 0.757 ± 0.034 | 0.755 ± 0.005 |
| 33 | 0.508 ± 0.006 | 0.724 ± 0.023 | 0.782 ± 0.025 | 0.753 ± 0.002 |
| 31 | 0.509 ± 0.006 | 0.741 ± 0.019 | 0.767 ± 0.020 | 0.754 ± 0.003 |
| 29 | 0.502 ± 0.010 | 0.724 ± 0.028 | 0.776 ± 0.029 | 0.750 ± 0.005 |
| 27 | 0.520 ± 0.006 | 0.692 ± 0.019 | 0.822 ± 0.017 | 0.757 ± 0.003 |
| 25 | 0.490 ± 0.006 | 0.726 ± 0.018 | 0.763 ± 0.016 | 0.745 ± 0.003 |
| 23 | 0.514 ± 0.016 | 0.670 ± 0.028 | 0.835 ± 0.035 | 0.753 ± 0.006 |
| 21 | 0.481 ± 0.006 | 0.709 ± 0.032 | 0.770 ± 0.030 | 0.740 ± 0.003 |
| 19 | 0.504 ± 0.009 | 0.624 ± 0.024 | 0.864 ± 0.024 | 0.744 ± 0.003 |
| 17 | 0.469 ± 0.009 | 0.683 ± 0.024 | 0.783 ± 0.022 | 0.733 ± 0.005 |

Supplementary Table S5. The results derived from different encoding schemes and their combinations were obtained by training the models on the CPLM 4.0 human training dataset and evaluating them on the human independent test dataset from CPLM 4.0.

| Encoding | MCC | SN | SP | ACC |
| --- | --- | --- | --- | --- |
| ALL | 0.623 | 75.68 | 86.13 | 81.19 |
| Embedding (Em) | 0.618 | 73.98 | 87.09 | 80.89 |
| One hot (OH) | 0.613 | 74.75 | 86.01 | 80.68 |
| AAindex (AA) | 0.620 | 74.96 | 86.44 | 81.01 |
| AA + Em | 0.621 | 74.69 | 86.78 | 81.06 |
| AA + OH | 0.621 | 74.66 | 86.84 | 81.08 |
| OH + Em | 0.619 | 74.38 | 86.88 | 80.97 |

Supplementary Table S6. Predictive performance of MMUbiPred compared to existing plant ubiquitination prediction model.

| **Predictor** | **ACC** | **F1-Score** |
| --- | --- | --- |
| MMUbiPred | **84.5** | **84.7** |
| UbiComb | 81.8 | 82.5 |
| Wang et al. | 73.3 | 74.9 |
| DeepUbi | 73.3 | 73.4 |
| Deep Ub | 67.4 | 68.7 |
| Ubisite | 59.6 | 68.1 |
| iUbiq-Lys | 56.3 | 67.1 |
| UbPred | 62.6 | 67.8 |

Supplementary Table S7. Predictive performance of MMUbiPred compared to hCKSAAP_UbSite model.

| **Predictor** | **AUC** |
| --- | --- |
| MMUbiPred | **0.823** |
| hCKSAAP_UbSite | 0.757 |

a
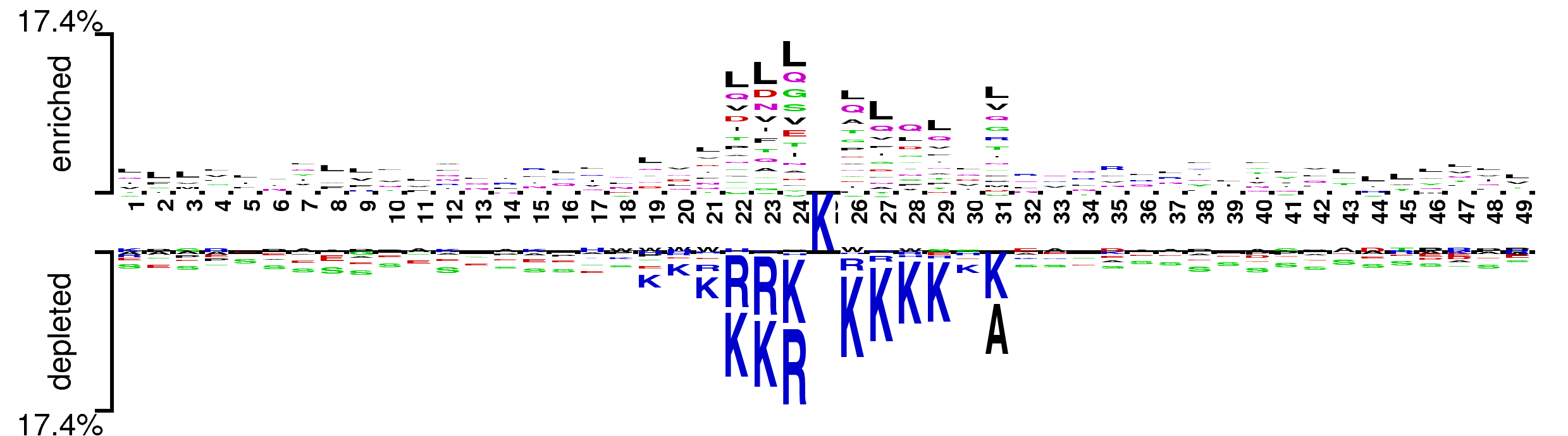


b
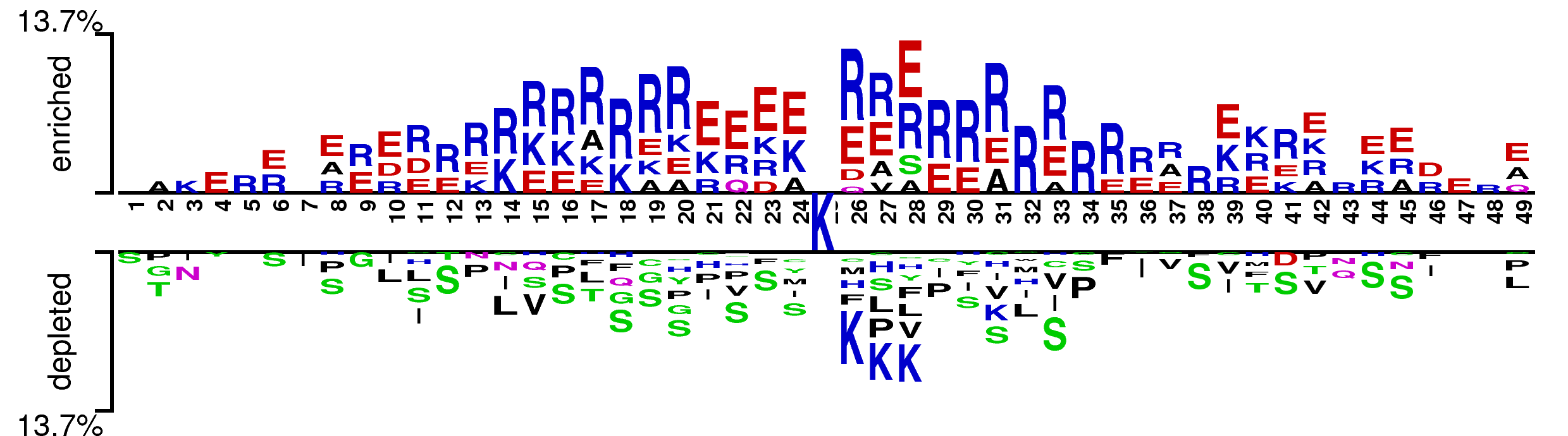


c
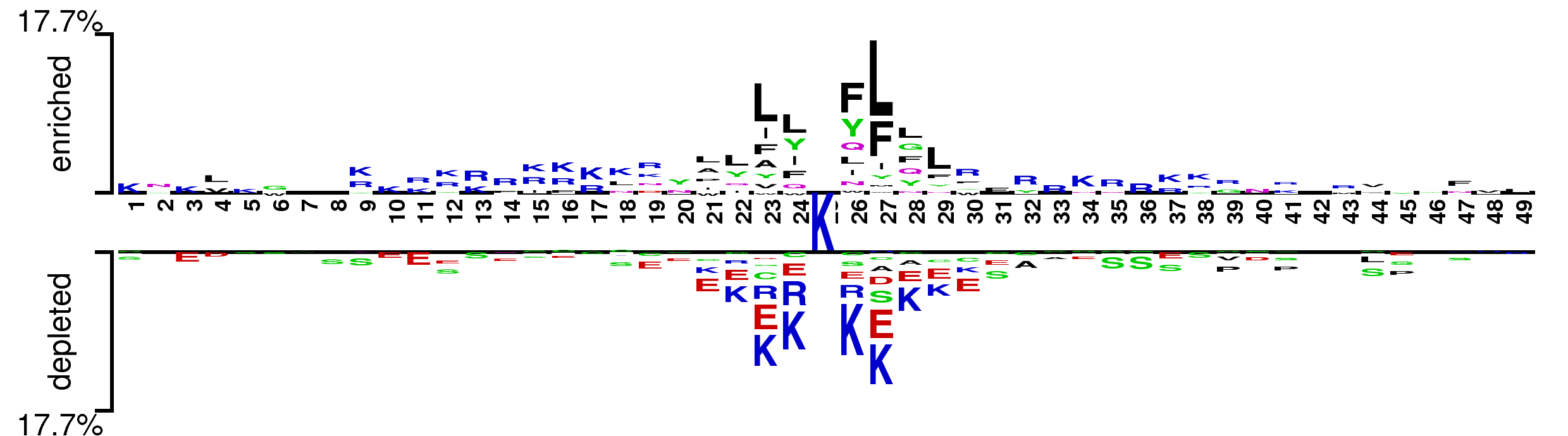


Supplementary Figure S1. Sequence logos of (a) general ubiquitination dataset (b) plant dataset and (c) human dataset. The significantly enriched or depleted residues at individual positions surrounding the ubiquitination sites are illustrated. These pictures were rendered using Two Sample Logo server (http://www.twosamplelogo.org/) with default settings.

| 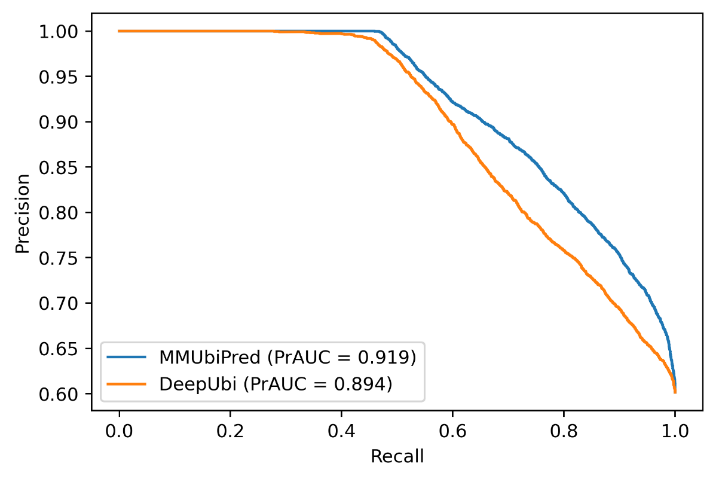 | 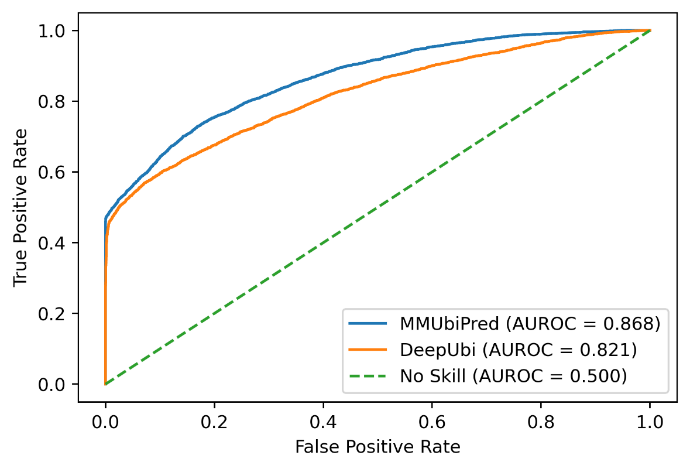 |
| --- | --- |
| (a) | (b) |

Supplementary Figure S2. (a) Precision Recall curve of MMUbiPred and DeepUbi, (b) ROC curve of MMUbiPred and DeepUbi
